# Intrajejunal Infusion of Levodopa/Carbidopa for Advanced Parkinson's Disease: A Systematic Review

**DOI:** 10.1002/mds.28595

**Published:** 2021-04-25

**Authors:** Taiji Tsunemi, Genko Oyama, Shinji Saiki, Taku Hatano, Jiro Fukae, Yasushi Shimo, Nobutaka Hattori

**Affiliations:** ^1^ Department of Neurology Juntendo University School of Medicine Tokyo Japan; ^2^ Neurodegenerative and Demented Disorders Juntendo University School of Medicine Tokyo Japan; ^3^ Home Medical Care System Based on Information and Communications Technology Juntendo University School of Medicine Tokyo Japan; ^4^ Department of Neurology Juntendo Nerima Hospital Tokyo Japan; ^5^ Research and Therapeutics for Movement Disorders Juntendo University School of Medicine Tokyo Japan

**Keywords:** Duodopa, intrajejunal, levodopa/carbidopa intestinal gel, Parkinson's disease, quality of life

## Abstract

Advanced Parkinson's disease is inconsistently defined, and evidence is lacking in relation to device‐aided therapies. To update existing reviews of intrajejunal infusion of levodopa/carbidopa (LCIG), we performed a literature search for relevant articles (to November 3, 2020) using PubMed supplemented by hand searching. Retrieved articles were categorized by relevance to identified research questions, including motor complications and symptoms; nonmotor symptoms; functioning, quality of life, and caregiver burden; optimal timing of treatment initiation and administration duration; discontinuation; and complications. Most eligible studies (n = 56) were open‐label, observational studies including relatively small patient numbers. LCIG consistently reduces OFF time and increased ON time without troublesome dyskinesia with varying effects regarding ON time with troublesome dyskinesia and the possibility of diphasic dyskinesia. More recent evidence provides some increased support for the benefits of LCIG in relation to nonmotor symptoms, quality of life, activities of daily living, and reduced caregiver burden. Patient age does not appear to significantly impact the effectiveness of LCIG. Discontinuation rates with LCIG (~17%–26%) commonly relate to device‐related issues, although the ability to easily discontinue LCIG may represent a potential benefit. LCIG may be a favorable option for patients with advanced Parkinson's disease who show predominant nonmotor symptoms and vulnerability to complications of other advanced therapy modalities. Larger, well‐controlled studies, including precise investigation of cost effectiveness, would further assist treatment selection. © 2021 The Authors. *Movement Disorders* published by Wiley Periodicals LLC on behalf of International Parkinson and Movement Disorder Society

Advanced Parkinson's disease (APD) is characterized by severe treatment‐related motor fluctuations and worsening motor symptoms, significant nonmotor symptoms, impaired quality of life (QoL), and prominent disability.^1^ Device‐aided therapies, which include intrajejunal infusion of levodopa/carbidopa intestinal gel (LCIG), deep brain stimulation (DBS), and continuous subcutaneous apomorphine infusion, are often initiated in APD for fluctuating response to conventional oral levodopa and adjunct agents (eg, catechol‐o‐methyl transferase inhibitors), severe dyskinesia, difficulty administering oral medications, or unacceptably high caregiver burden. An intestinal gel consisting of entacapone combined with LCIG has also been studied recently as a means of increasing levodopa plasma concentration. Device‐aided therapy uptake has increased recently,^2^ possibly related to protocols and insights from experienced practitioners, which helps less familiar clinicians incorporate these options.^3^ Each device‐aided therapy has merits and drawbacks, necessitating careful consideration of their appropriate selection and use, including attention to individual patient and caregiver needs.

A previous systematic review by Wirdefeldt et al.^4^ provided a comprehensive assessment of the effects of LCIG on motor symptoms and dyskinesia, nonmotor symptoms, QoL, and safety. This review concluded that moderately high evidence exists that LCIG reduces fluctuating motor symptoms and improves QoL while pointing out issues with the quality of evidence overall and especially for reduction of nonmotor symptoms. Other systematic and narrative reviews have provided practical recommendations on LCIG, including patient selection, efficacy and tolerability profiles, and contraindications.^5^, ^6^, ^7^, ^8^ Despite this, several issues remain incompletely resolved. These include the effect of LCIG on specific motor complications and symptoms, specific nonmotor symptoms (especially impulse control disorders [ICDs] and sleep), activities of daily living (ADL), QoL, and caregiver burden. The optimal duration of administration and timing of initiation in relation to disease progression and patient age also require further attention. Finally, detailed information on discontinuation has been lacking. Physician and patient focus group discussions suggest that evidence‐based decision‐making regarding APD is suboptimal and highlight the importance of individual patient preferences.^9^ Consequently, this review aims to address these issues with a focus on more recent evidence.

## Materials and Methods

### Search Strategy

Relevant human studies were searched using PubMed (up to November 3, 2020) with the following search terms: “parkinson disease,” “parkinson's,” “levodopa,” “carbidopa,” and “infusion.” Boolean operators combined search terms using the “OR” operator within categories and the “AND” operator for terms between categories.

### Eligibility Criteria

Articles were screened for relevance to LCIG and to: (1) motor complications and individual symptoms of APD; (2) nonmotor symptoms; (3) functioning, QoL, and caregiver burden; (4) optimal timing of treatment initiation and administration duration; (5) discontinuation; and (6) common complications. Articles comprising nonclinical design (eg, pharmacokinetic studies), no reportable relevant data, published >10 years ago or interim results, non‐English language, small patient numbers (n ≤10), nonstudy publication types (eg, narrative reviews, letters to the editor), or studies of different disease states were excluded. Citations from recent reviews were reviewed to source additional relevant articles.

### Quality Assessment

Quality was assessed using a five‐point rating scale modified from the Oxford Centre for Evidence‐based Medicine (available at: https://www.cebm.ox.ac.uk/resources/levels-of-evidence/ocebm-levels-of-evidence) for ratings of individual studies and based on study type: 1 = properly conducted randomized clinical trial, systematic review with metaanalysis; 2 = well‐designed controlled trial without randomization, prospective comparative cohort trial; 3 = case–control studies, retrospective cohort studies; 4 = case series, cross‐sectional studies; and 5 = case reports, opinion of respected authorities. Studies were also assessed in terms of susceptibility to bias and other limitations as reported in the publication or assessed by the authors. However, no quantitative analyses of treatment effects were conducted.

## Results

**FIG. 1:**
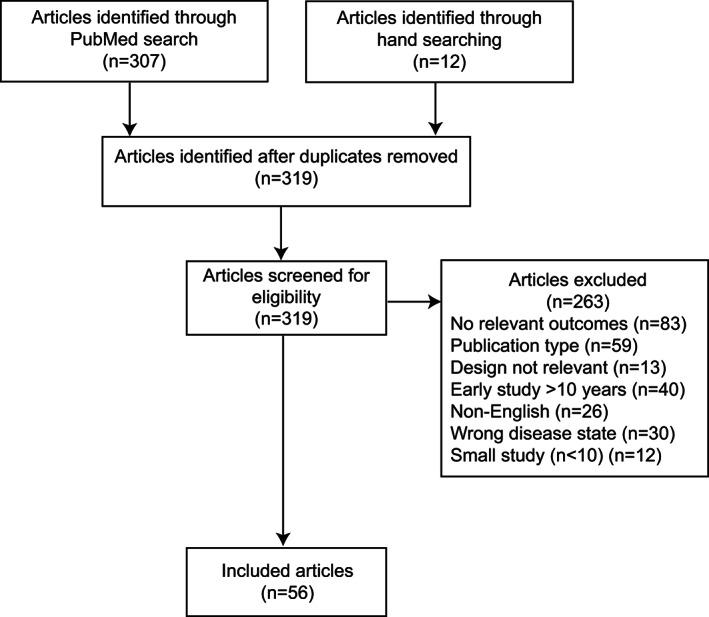
Disposition of studies.

Figure 1 shows the search process and results, including reasons for article exclusion. After screening and eligibility assessment, 56 core articles were included (Table S1), comprising 6709 enrolled patients; most involved small patient populations (median, 54 patients) with study size generally greater in more recent studies. Studies generally occupied the middle range of the quality rating scale (rating scale, 1 [n = 1], 2 [n = 2], 3 [n = 47], 4 [n = 5], and 5 [n = 1]). Many studies were deemed to be at moderate or high risk for potential bias and other limitations, which reflects the observational nature of almost all included studies and often small patient numbers. Overall, there were 23 open‐label, prospective observational studies; 11 open‐label, retrospective observational studies; 7 post hoc analyses; 4 case series or reports; 3 cohort studies; and 1 each of the following study types: open‐label mixed prospective/retrospective observational study, postmarketing surveillance study, clinician survey, cross‐sectional epidemiological study, randomized controlled study, open‐label extension of a double‐blind, pivotal study, combined retrospective, cross‐sectional survey and longitudinal case–control study, and a systematic literature review plus metaanalysis.

### Effects of LCIG on Motor Complications and Individual Symptoms

#### Effects of LCIG on Dyskinesia

The effects of LCIG on motor complications were assessed in a US pivotal 12‐week double‐blind study of patients with APD of approximately 10 to 12 years in duration,^10^ which compared LCIG with oral levodopa/carbidopa, and a 52‐week open‐label extension study.^11^ Recent observational studies demonstrate that LCIG reduces OFF time and increases ON time with no disabling dyskinesia (ie, without troublesome dyskinesia) typically by approximately 4 hours for each endpoint over mean administration periods of up to 10 years (Table 1).^12^, ^13^, ^14^, ^15^, ^16^, ^17^, ^18^ A metaanalysis supports these results but showed no reduction in ON time with troublesome dyskinesia.^19^ Regarding the effect of LCIG on troublesome dyskinesia, recent studies, including a post hoc analysis of the pivotal GLORIA registry trial, suggest LCIG has greater benefits in reducing dyskinesia among patients experiencing greater dyskinesia episodes at baseline,^19^, ^20^, ^21^ possibly because of patient characteristics and the altered mode of delivery, which has been suggested to achieve more constant blood levels^22^ and an increase in the threshold for levodopa‐induced dyskinesia.^23^ In a comparative study of LCIG and DBS, both procedures improved mean waking OFF time in association with improvements in Unified Parkinson's Disease Rating Scale Part II (UPDRS II), UPDRS III, and UPDRS IV,^24^ and LCIG has also been shown to be potentially beneficial in DBS patients who have refractory symptoms.^25^

**TABLE 1:** Reductions in OFF time and increases in ON time without troublesome dyskinesia: results from recent observational studies

Reference	Aim	Design and observation period	Patient population	OFF time reduction^a^	ON time^b^ increase^a^	Comments
Antonini et al. (2017)^12^	Evaluate LCIG in patients with APD under routine clinical care	Registry, 24 mo	Routine use, 375 enrolled, 258 completed	4.1		Final results of GLORIA registry study
Bohlega et al. (2015)^13^	Report on Middle Eastern single‐center experience with LCIG	OL, prospective median (range) follow‐up period was 48.5 (11–83) mo	Routine use, 20 patients enrolled, 18 patients included in analysis	3.8		Improvements in motor complications associated with improvements in UPDRS III and QoL
De Fabregues et al. (2017)^14^	Assess long‐term safety and effectiveness of LCIG in patients with APD	OL, prospective up to 10 years	Severe fluctuations, 37 enrolled over course of study	4.9 (3 mo [n = 37]) to 6.3 (9 years [n = 2])		Substudies reported on QoL, health status, and caregiver burden scales
Fernandez et al. (2018)^15^	Report long‐term safety and efficacy outcomes from an OL phase 3 treatment program	OL, extension of double‐blind study and safety/efficacy, 52–54 weeks and 5+ years ongoing OL extension	APD with motor fluctuations, 262 continuing patients	~4	~4	Reduction in ON time with troublesome dyskinesia initially noted but not maintained during extension period
Juhasz et al. (2017)^16^	Determine whether UPDRS and UDysRS could detect improvement in ADL	OL, prospective 12 mo	Registry patients with severe fluctuations, 34 consecutive patients	4.5 (median)	6.8 (median)	Confirmed that LCIG treatment improves experiences of daily living (see text)
Lopiano et al. (2019)^17^	Assessed impact of LCIG on motor and nonmotor symptoms, QoL, and caregiver burden	PMS, retrospective (LCIG 1–7 years), prospective (LCIG <1 year)	APD with motor fluctuations, 137 evaluable	~4 (50% reduction in UPDRS IV item 39)		Improvements in ADL also noted
Murata et al. (2018)^18^	Reported efficacy and safety of LCIG over 52 weeks in Japanese, Taiwanese, and Korean patients	OL extension study (median LCIG duration, 408 days)	PD with severe motor fluctuations, 28 patients enrolled, 27 completed >52 weeks treatment	4.6	5.0	Improvements in various QoL domains also noted

^a^ Hours per day (mean unless otherwise stated).

^b^ Without troublesome dyskinesia.

LCIG, levodopa/carbidopa intestinal gel; APD, advanced Parkinson's disease; OL, open label; UPDRS, Unified Parkinson's Disease Rating Scale; QoL, quality of life; UDysRS, Unified Dyskinesia Rating Scale; ADL, activities of daily living; PMS, postmarketing study.

Diphasic dyskinesia associated with LCIG was specifically reported in a small number of articles. In one study (n = 33), four patients (12.2%) with disease duration of 14 to 21 years and motor complications for 8 to 10 years experienced disabling diphasic dyskinesia after LCIG initiation.^26^ Patients were managed by increasing morning and continuous LCIG flow generally followed by combination dopamine agonists and sustained‐release levodopa formulations during bedtime. A more recent retrospective cross‐sectional survey of patients with atypical phenomenology (eg, biphasic‐like dyskinesias) reported atypical (long‐lasting) biphasic, biphasic‐like (continuous), or mixed (peak‐dose and continuous biphasic) dyskinesias after levodopa infusion in 30 of 208 patients (14.4%).^27^ Increasing the dopaminergic load improved biphasic‐like dyskinesia in about half of affected patients, whereas mixed dyskinesias had the worst outcome and a high discontinuation rate.

#### Effects of LCIG on Axial Symptoms and Gait

Axial symptoms (eg, dysarthria, dysphagia, dyspnea, and balance/walking difficulties) are largely mediated by nondopaminergic pathways and are difficult to treat with levodopa.^28^ An open‐label study suggested that LCIG improves freezing of gait (FOG) and other gait/balance parameters,^29^ as confirmed more recently in larger populations^30^, ^31^ and during periods of over 4 years in patients with disease duration of 18 years at end of follow‐up.^32^ Over long‐term follow‐up, axial scores and FOG gradually deteriorated, although milder disease severity predicted better axial outcomes, whereas lower baseline axial response to levodopa predicted a worse outcome in terms of FOG and lower long‐term ADL independence.

### Effects of LCIG on Nonmotor Symptoms

Nonmotor symptoms in APD, including neuropsychiatric symptoms, cognitive dysfunction, hyposmia, pain, fatigue/sleep disturbances, autonomic dysfunction, and weight loss, have a major impact on QoL and also increase caregiver burden.^33^, ^34^, ^35^ The effect of dopaminergic therapy on nonmotor symptoms correlates with motor response in APD,^36^ highlighting the importance of optimizing dopaminergic therapy to reduce the impact of nonmotor and motor symptoms.

Final results of large registry and open‐label studies confirm LCIG improves numerous nonmotor symptoms as evidenced by significant reductions in total Non‐Motor Symptoms Scale (NMSS), individual domain items, and other measures.^12^, ^16^, ^37^, ^38^, ^39^, ^40^, ^41^ Individual domains that consistently showed improvement in two studies of patients with a mean disease duration of 12.5 to 13.9 years and mean total NMSS of 69.2 to 95.5 included “sleep/fatigue,” “gastrointestinal tract,” and “miscellaneous.”^37^, ^38^, ^39^ In a post hoc analysis of the 24‐month GLORIA registry trial (n = 375) of LCIG in routine clinical practice,^39^ the median change in NMSS total score for the overall population (−18.0, n = 170) was positively correlated with the baseline NMSS score. Further, baseline NMSS >80 was associated with the greatest proportion of responders as assessed by a decrease in NMSS ≥30 (47% vs ≤28% for other baseline NMSS groups).

#### Effects of LCIG on Sleep

Large‐scale patient surveys suggest sleep disturbances are the most disruptive nonmotor complication of APD.^42^ Improvements in the Parkinson's disease sleep scale (PDSS) score were noted in a retrospective, open‐label study (n = 14, age: 67.0 ± 11.5 years, disease duration: 12.9 ± 4.8 years) of LCIG, which found that the PDSS improved by 14% (*P* = 0.008) from baseline after mean follow‐up of approximately 24 months.^43^ An analysis of 12 LCIG‐treated patients (age: 71.7 ± 4.8 years, disease duration: 16.9 ± 4.4 years) support these results, showing PDSS scores improved by 34.0% (*P* = 0.005), disturbed sleep improved by 31.7% (*P* = 0.005), and motor symptoms at night improved by 27.9% (*P* = 0.017) after 2 to 4 months of treatment.^44^ In addition, an open‐label, prospective trial (n = 15, age: 67.0 ± 6.0 years, disease duration: 12.0 ± 5.0 years), in which patients reported sleep problems (PDSS‐2 total score ≥ 11), revealed that only seven patients reported sleep problems (*P* = 0.021) 1 year after LCIG initiation, in association with clinically meaningful total PDSS‐2 reductions.^16^ Finally, a subanalysis of the GREENFIELD study found a significant improvement in mean PDSS‐2 from baseline (25.0 ± 10.4) to visit 3 (22.7 ± 10.1, *P* < 0.01).^17^ Thus, LCIG generally improves sleep problems measured by PDSS probably because of improvement of nocturnal motor symptoms. Improvements in the NMSS sleep/fatigue domain were linked to QoL improvements in a post hoc analysis of the GLORIA registry study.^39^

#### Effects of LCIG on ICD

ICD (eg, compulsive gambling, binge eating) are common complications of dopamine replacement therapy associated with demographic and clinical risk factors (eg, younger age at onset, more frequent dyskinesia) that greatly impair patient functioning and QoL and are especially disruptive for caregivers.^45^

An observational study (n = 19) showed that six of eight LCIG‐treated patients with ICD had resolution of their disorder,^46^ while a larger, more recent, prospective, observational study (n = 62, age: 72.7 ± 7.0 years, disease duration: 13.5 years) with 6‐month follow‐up found LCIG progressively and significantly improved ICD, including a 64% reduction in the Questionnaire for Impulsive‐Compulsive Disorders in Parkinson's Disease–Rating Scale score.^47^ The GREENFIELD study (n = 145) showed significant improvements compared with baseline in the total Questionnaire on Impulsive Disorders (QUIP‐RS) score and, in patients receiving LCIG for <1 year before the first visit, the QUIP‐RS subitems sexual behavior, eating, and medication use.^17^ These improvements were considered to be caused by reduction of dopamine agonists.

### Effects of LCIG on ADL, QoL, and Caregiver Burden

Depression, disability, impairments in physical function, especially FOG and postural immobility, and sleep disturbances are important determinants of QoL in PD, whereas specific motor symptoms (eg, tremor) have relatively minor influence.^42^, ^48^, ^49^ A considerable number of studies indicate that LCIG positively affects QoL (Table 2).^12^, ^13^, ^14^, ^16^, ^17^, ^37^, ^38^, ^40^, ^50^, ^51^, ^52^ Correlation analyses in one open‐label, observational, prospective study (n = 12; age: 73.8 ± 7.9 years; disease duration: 12.8 ± 5.0 years) did not show significant relationships between clinical conditions and QoL or between levels of anxiety, depression, and burden with caregiver QoL, possibly because of small patient numbers.^50^ In a post hoc analysis of the GLORIA registry study (n = 233), moderate positive correlations were found between the NMSS total score and Parkinson's Disease Questionnaire (PDQ)‐8 total score (r = 0.46, *P* < 0.0001), improvements of the sleep/fatigue domain and PDQ‐8 total score (r = 0.32, *P* = 0.0001), and the mood/cognition domain and PDQ‐8 total score (r = 0.37, *P* < 0.0001) during a 2‐year period.^39^ Most recently, a prospective, open‐label study of 12 patients followed for 6 months reported that LCIG improved individual QoL in both patients and caregivers as assessed by the Schedule for the Evaluation of Individual Quality of Life Questionnaire.^51^ This improvement was observed despite no significant change in health‐related QoL, as assessed by the PDQ‐39, and was thought to be influenced by psychological factors given that family and relationships were rated highly by both patients and caregivers.

**TABLE 2:** Effects of LCIG on measures of QoL: results from recent observational studies

Reference	Aim	Design/observation period	Patient population	Effects on QoL
Antonini et al. (2017)^12^	Evaluate LCIG in patients with APD under routine clinical care	Registry, 24 mo	Routine use, 375 enrolled, 258 completed	PDQ‐8 significantly improved at every study visit (change at last visit: −5.3 ± 20.7, *P* < 0.001, n = 205)
Bohlega et al. (2015)^13^	Report single‐center experience of LCIG in Middle East	OL, prospective	20 consecutive patients with APD with motor fluctuations and nonmotor symptoms	Mean PDQ‐8 improved from 23.2 ± 4.4 before LCIG to 8.0 ± 3.5 after 6 mo (*P* < 0.001)
Cáceres‐Redondo et al. (2014)^37^	Investigate the long‐term motor and cognitive outcomes of LCIG in APD	OL, retrospective, >24 mo	29 patients enrolled, 16 completed 24‐month treatment	Mean PDQ‐39 improved from 84.2 ± 18.7 at baseline to 74.3 ± 21.3 after a mean period of 32.2 ± 12.4 mo (*P* < 0.05)
Ciurleo et al. (2018)^50^	Evaluate the impact of LCIG on PD patients and caregivers, and their QoL	OL, prospective	Routine use, 12 consecutive patients	Mean PDQ‐39 improved from 54.8 ± 6.0 at baseline to 13.3 ± 1.8 at 6 mo (*P* = 0.002)
De Fabregues et al. (2017)^14^	Assess long‐term safety and effectiveness of LCIG in patients with APD	OL, prospective up to 10 years	Severe fluctuations, 37 enrolled over course of study	Mean PDQ‐39 improved from 56.9 ± 11.4 at baseline to 35.5 ± 18.8 at 1 year (*P* = 0.018, n = 9)
Ehlers et al. (2020)^51^	Observe effects of LCIG on iQoL in patients and caregivers	OL, prospective, 6 mo	12 patients with severe disease who received LCIG	Patient and caregiver iQoL improved, especially in relation to family, relationships, health, independence; improvements also seen in various nonmotor symptoms and caregiver burden
Juhasz et al. (2017)^16^	Determine whether UPDRS and UDysRS could detect improvement in ADL	OL, prospective 12 mo	Registry patients with severe fluctuations, 34 consecutive patients	Mean PDQ‐39 improved from 38.5 ± 14.9 at baseline to 29.6 ± 13.6 at 1 year (*P* = 0.003)
Krüger et al. (2017)^38^	Assess the effect of LCIG on ADL, motor and nonmotor symptoms, and QoL in patients with APD	PMS, prospective	Routine use, 64 patients	Mean PDQ‐8 improved significantly at each visit (3, 6, 12 mo, final for patients with missing data)
Lopiano et al. (2019)^17^	Assessed impact of LCIG on motor and nonmotor symptoms, QoL, and caregiver burden	PMS, retrospective (LCIG 1–7 years), prospective (LCIG <1 year)	APD with motor fluctuations, 137 evaluable	Mean PDQ‐39 improved from 72.3 ± 23.8 at baseline to 67.3 ± 26.4 at visit 3 (year 2) (*P* < 0.05)
Standaert et al. (2018)^41^	Assessed efficacy of LCIG on motor and nonmotor symptoms, QoL, and safety	OL, prospective, 60‐week	38 patients with successful PEG‐J who received levodopa infusion	Least square mean PDQ‐39 improved from 34.7 ± 13.0 at baseline by −10.2 ± 2.6 by week 60 (*P* < 0.001)

LCIG, levodopa/carbidopa intestinal gel; QoL, quality of life; APD, advanced Parkinson's disease; PDQ, Parkinson's Disease Questionnaire; OL, open label; iQoL, individual quality of life; UPDRS, Unified Parkinson's Disease Rating Scale; UDysRS, Unified Dyskinesia Rating Scale; ADL, activities of daily living; PMS, postmarketing study; PEG‐J, percutaneous endoscopic transgastric jejunostomy.

ADL have been assessed in open‐label studies of LCIG, mostly via UPDRS II scores and the Schwab and England Activities of Daily Living scale. In one study of 37 patients, the median Schwab and England Activities of Daily Living score increased significantly from 50 at pretreatment to 80 after 3 months of LCIG treatment (*P* < 0.001),^14^ and patients went from being dependent and having difficulties with all activities to being completely independent in most chores. Similarly, UPDRS II scores increased significantly in several other studies, including at periods as early as 3 months after baseline and over longer periods of follow‐up.^15^, ^17^, ^21^, ^38^ Hence LCIG may improve ADL along with improvements in motor complications. LCIG has also recently shown beneficial effects on workplace participation in a retrospective study from three centers in Sweden and Denmark in which most patients could perform workplace activities to the same extent or more 5 years after treatment introduction.^53^

Few studies have assessed caregiver burden of patients receiving LCIG. The effect of LCIG on caregiver burden has been commonly assessed by the Zarit Burden Interview (ZBI) and depression or anxiety indices. Over long‐term follow‐up in one observational study of LCIG (n = 37), ZBI scores significantly improved by 20% at 3 months in 9 patients (*P* = 0.042), although improvement was lower and nonsignificant after 1 year.^14^ A similar open, prospective study (n = 12, age: 73.8 ± 7.9 years) also found improvements in caregiver burden, anxiety, depression, and QoL.^50^ In a more recent cross‐sectional, epidemiological study conducted among 126 patients (age: 69.3 ± 8.0 years) and their caregivers (age: 57.9 ± 12.9 years), ZBI scores tended to be lower among LCIG‐treated patients than those treated with continuous subcutaneous apomorphine infusion or standard of care.^54^ LCIG appears to have a favorable impact on caregiver burden,^55^, ^56^, ^57^ although more well‐designed prospective studies are needed.

### Optimal Timing of Treatment Initiation and Diurnal Administration Duration

#### Optimal Age Range for LCIG Initiation

Previous recommendations suggested that age does not affect LCIG efficacy despite being preferred in older, frail patients because of improved tolerability^58^ and a greater ability to discontinue treatment if necessary. However, studies to date provide conflicting conclusions and suggest that earlier LCIG initiation in younger patients and those with shorter disease duration may improve outcomes.^59^ One prospective, open‐label study (n = 28, age: 67.6 ± 6.1 years, disease duration: 15.5 ± 4.0 years) of 24‐month LCIG treatment concluded that younger patients had better outcomes in terms of OFF time response (>50% improvement in UPDRS item 39).^29^ However, although statistically significant (*P* = 0.039), the difference in mean age was <2 years between responders and nonresponders, and patient numbers were low (12 and 5 patients, respectively). A larger, retrospective analysis (n = 113, median age: 65 years, median disease duration: 12 years) of LCIG in a real‐world setting found men ≤60 years old had greater improvements in mean OFF time (89.8%) than men >60 years old (82.6%), although this difference was not observed in women (81.9% vs 83.2% for ≤60 vs >60 years old, respectively).^60^ Further, analysis of the GREENFIELD study found that younger age correlated with slightly more disabling complications, and shorter disease duration (<13 years) led to better outcomes for motor complications and ADL.^17^ These results are partly supported by a retrospective analysis of 177 patients who reported significantly greater reduction in OFF time among patients with disease duration less than 10 years than in patients with disease duration of 10 years or greater (−38% vs −29%, *P* = 0.021).^61^ In contrast, a recent analysis of a 54‐week phase III study (n = 324) that examined responder and nonresponder characteristics suggested that age and disease duration do not influence LCIG response.^40^ Overall, effects of age or duration of disease on LCIG outcomes appear modest, despite evidence suggesting shorter disease duration may improve outcomes.

#### Optimal Duration of Diurnal Administration with LCIG

Few studies have compared the approved 16‐hour LCIG infusion with 24‐hour infusions, which may be used if medically justified. A retrospective case series of 21 patients with mean age of 69 to 70 years and disease duration of approximately 16 to 18 years found transition to 24‐hour infusion improved sleep quality but increased levodopa dosage (15% ± 24% over 32 months).^62^ A more recent retrospective review of 35 patients who received 24‐hour infusion for a variety of indications (mainly FOG and/or troublesome dyskinesia unresponsive to 16‐hour infusion) found that the incidence of de novo adverse events was not significantly increased by the longer infusion period.^63^ In contrast, total UPDRS IV and complexity of motor fluctuations subscore were significantly reduced after approximately 1 year with 24‐hour infusion compared with 16‐hour infusion. Despite these suggested benefits, current evidence suggests 24‐hour LCIG infusions should remain limited to certain situations (eg, unresponsive motor complications, severe nocturnal disability). Larger studies are needed to determine benefits of diurnal infusion versus potential drawbacks, apart from expected increases in dosage and medication costs.

### Discontinuation

Previous evidence summaries have highlighted the complexity of LCIG discontinuation, including a lack of consensus regarding the effects of age and other variables.^7^ Real‐world use studies involving large patient numbers and long‐term follow‐up periods provide the most robust evidence regarding the rate and characteristics of LCIG discontinuation (Table 3).^15^, ^66^, ^67^, ^69^, ^70^, ^71^ In an integrated safety analysis from four prospective multicenter clinical trials, discontinuations related to adverse events were seen in 17% of 412 patients enrolled in open‐label studies.^66^ A retrospective review of 905 patients included in a survey of Italian specialists on clinical and practical aspects of LCIG therapy reported an overall discontinuation rate of 25.7%, with 9.5% of discontinuations in the first year.^70^ This annual rate is similar to that of an open‐label phase III study of 262 patients with a disease duration of approximately 10 years who completed a 12‐week double‐blind study and 52‐week open‐label extension or a separate 54‐week open‐label study.^15^ More recently, a long‐term, retrospective, longitudinal study of 105 consecutive patients with a total mean levodopa‐equivalent daily dose of 1148 mg reported a comparable discontinuation rate of 22% after a mean treatment duration of 3.0 ± 2.6 years.^64^ Analysis of mortality predictors showed that mortality was not significantly different between patients who discontinued and those who continued LCIG, nor did it correlate with the number of serious adverse events, whereas lower Mini‐Mental State Examination score at the start of LCIG did predict higher mortality. Another retrospective study of 204 patients observed over a 10‐year period reported a highly similar discontinuation rate (21%), which was linked to a more severe clinical picture in terms of motor symptoms and cognitive decline, greater incidence of peripheral neuropathy, and shorter disease duration at baseline.^65^ Indeed, approximately half of the patients who dropped out were receiving 24‐hour infusion. A slightly lower discontinuation rate (18.4%) was noted in a recent retrospective cohort study of 98 patients receiving long‐term LCIG (age: 66.2 ± 8.2 years; disease duration: 12.3 ± 5.4 years), which also conversely reported that shorter disease duration at baseline was associated with a lower rate of discontinuation.^68^ Discontinuation rates were also noted to be lower among patients receiving LCIG monotherapy compared with LCIG polytherapy in a post hoc analysis of the GLORIA registry study.^21^ Complications related to the device, lower than expected efficacy, and death tended to be the most consistently noted events leading to discontinuation throughout relevant studies.^20^, ^64^, ^65^, ^66^, ^68^, ^70^

**TABLE 3:** Discontinuation rates with LGIC: results from recent studies

Reference	Aim	Design/observation period	Patient population	Discontinuation data	Comments
Artusi et al. (2020)^64^	Analyze mortality and its predictors with LCIG over 10 years	OL, retrospective, longitudinal, >10 years	91 patients with mean disease duration 13.0 ± 4.3 years	20 (22%) patients discontinued over mean period of 3.0 ± 2.6 years	Main causes of discontinuation: neuropathy (45%), late‐stage PD (20%), no clinical benefit (10%), abdominal pain (10%), PEG‐J displacement/surgical abdominal complications (10%), poor compliance (5%)
Constantin et al. (2020)^65^	Analyze causes of discontinuation over 10 years	OL, retrospective, over 10 years	204 patients	43 (21%) discontinued over mean duration of 21.6 mo	Main causes of discontinuation: AEs (24%), lack of efficacy (3%), withdrawn consent (6%), administrative reasons (<1%), and protocol violations (<1%)
Fernandez et al. (2018)^15^	Report long‐term safety and efficacy outcomes from an OL phase 3 treatment program	OL, extension of DB study and safety/efficacy, 52–54 weeks and 5+ years ongoing OL extension	APD with motor fluctuations, 262 continuing patients	89 (34%) patients discontinued LCIG for any reason	Main causes of discontinuation: death, poor compliance, acute psychosis, peripheral neuropathy, device complications
Lang et al. (2016)^66^	Summarize safety data from 4 studies	Integrated data analysis from 4 prospective, phase 3 studies	395 patients (PEG‐J), 412 patients (all OL)	OL patients: 72 (17%) patients discontinued LCIG due to an AE overall	Main causes for discontinuation: complication of device insertion (2.4%), death (1.2%), abdominal pain (1.0%), pneumonia (1.0%), myocardial infarction (0.7%), and fall (0.7%)
Lew et al. (2015)^67^	Compare 2 LCIG dosing regimens from phase 3 studies	Data taken from OL and DB trials	OL, 354 patients (324 switched to PEG‐J by week 4) DB, 37 patients	30 (8%) patients discontinued during NJ phase mainly due to withdrawal of consent (n = 12 patients), protocol violation (n = 7 patients)	Discontinuations not due to procedure or device were low (2.2% and 2.7% in the OL and double‐blind studies, respectively)
Moes et al. (2020)^68^	Explore baseline predictors of time to discontinuation of LCIG	Retrospective cohort study, mean 2.6 years	98 patients with mean disease duration 12.3 ± 5.4 years	18 (18.4%) patients discontinued after mean duration of 7.8 years	Main causes for discontinuation: device related (5.1%), lack of effect (7.1%), switch to other therapy (5.1%)
Poewe et al. (2019)^69^	Compare the effectiveness and safety of LCIG monotherapy vs polytherapy	Post hoc analysis of 24‐month, multinational observational registry (GLORIA)	208 patients on stable regimens (LCIG monotherapy, n = 80; levodopa monotherapy, n = 47; LCIG polytherapy, n = 81)	Stable LCIG monotherapy group had the lowest numerical dropout rate (26% at month 24), and the stable LCIG polytherapy group had the highest numerical dropout rate (33% at month 24)	Patients with adverse drug reactions leading to discontinuation were low: LCIG monotherapy (6%), levodopa therapy (9%), LCIG polytherapy (6%)
Sensi et al. (2017)^70^	Report results of Italian survey	Retrospective review of survey results	905 patients included	233 (25.7%) patients discontinued overall	Main causes of discontinuation: caregiver noncompliance (15.0%), worsening cognitive decline (14.1%), death (11.6%), stoma infection (11.5%)
Zibetti et al. (2014)^71^	Report 7‐year experience of patients treated with LCIG	OL, prospective, observational 7 years	59 consecutive patients	11 (19%) patients discontinued after a time lag of 19.3 ± 14.9 (range 3–56) mo	Causes of discontinuation: cerebral hemorrhage (n = 1), traumatic brain injury (n = 1), device management, cognitive decline (n = 4), inefficacy (n = 2), polyneuropathy (n = 1), weight loss (n = 2)

LCIG, levodopa/carbidopa intestinal gel; OL, open label; PD, Parkinson's disease; PEG‐J, percutaneous endoscopic transgastric jejunostomy; AE, adverse event; DB, double‐blind; APD, advanced Parkinson's disease; NJ, nasojejunal.

#### Complications of LCIG

Complications of LCIG are generally divided into device‐ or procedure‐related issues and those arising from levodopa/carbidopa administration. Device‐related complications were commonly noted in the literature reviewed and especially included dislocation, migration, occlusion, and kinking of tubing, deterioration of connectors, and accidental removal.^10^, ^12^, ^15^, ^20^, ^21^, ^24^, ^37^, ^43^, ^61^, ^66^, ^67^, ^69^, ^72^ Procedure‐related complications were typically less common, but prominent among these were stomal and other infection, abdominal pain, granuloma formation, and pseudoperitoneum.^15^, ^16^, ^24^, ^25^, ^37^, ^38^, ^66^, ^67^, ^72^, ^73^

Levodopa‐related complications associated with LCIG noted in the literature included hallucinations, confusion, psychotic disorders, insomnia, falls, and dyskinesia.^15^, ^16^, ^21^, ^24^, ^25^, ^38^, ^63^, ^66^, ^67^, ^69^, ^72^, ^73^, ^74^ In addition, weight loss and peripheral neuropathy were noted throughout the literature and have been specifically assessed in several studies. In two separate observational studies of patients with long‐term disease duration of approximately 15 years, mean weight losses of 3.0 kg over 32 months and 7.6 kg over 52 months were reported.^37^, ^75^ Another long‐term study reported similar levels of weight loss, and although almost half of all patients had weight loss ≥7.0%, only 3.8% of patients had serious weight loss.^15^ One open‐label, observational study found that the extent of weight loss correlated significantly with the percentage of the waking day spent with dyskinesia, whereas nutritional status correlated with motor symptom severity, dysphagia, and levodopa‐equivalent daily dose.^15^ Regarding peripheral neuropathy, an earlier prospective observational study reported more severe neurographic abnormalities in 15 patients who had received LCIG for an average of 736 ± 420 days than those observed in matched controls who received conventional treatment.^76^ Among LCIG‐treated patients, the degree of neuropathic change correlated with weight lost since therapy initiation and levodopa dose. A subsequent long‐term prospective evaluation of 33 consecutive patients treated with LCIG also observed that higher levodopa‐equivalent dose, as well as homocysteine levels, were found in patients with chronic peripheral neuropathy.^77^ However, vitamin B_1_/B_12_ supplementation led to clinical improvement and/or substantial stabilization after further evaluation. Another prospective pilot study, in which patients received early and continuous vitamin B supplementation from initiation of LCIG, reported a relatively low rate of new cases of peripheral neuropathy (19%) after a mean (range) follow‐up period of 42.4 (24–72) months.^78^ The authors suggested that chronic vitamin B supplementation may stabilize new‐onset and preexisting peripheral neuropathy, even if it is not able to fully prevent new cases.

## Discussion

The findings of this review corroborate those of previous reviews and literature‐based recommendations, although more recent data have clarified issues that may affect device‐aided therapy selection. In Europe, LCIG has been available since approval in 2004, whereas the US Food and Drug Administration provided approval in 2015. More recent evidence suggests LCIG is associated with benefits for nonmotor symptoms (particularly sleep and ICD), QoL, and functional activity, which have become prioritized as the need for device‐aided therapies expands. A randomized 26‐week study comparing LCIG with optimized medical treatment with regard to nonmotor symptoms is also nearing completion (ClinicalTrials.org: NCT02549092), which will assess changes in the PDSS‐2 total score and NMSS total score in addition to multiple secondary outcomes, including impulsivity, depression, and pain. The mechanisms by which LCIG improves sleep and fatigue are potentially harder to understand than with DBS but may relate to treatment “simplification.”^16^, ^79^ Larger studies are needed to clarify the sleep benefits of LCIG, but it is reasonable to propose that LCIG does not worsen sleep quality and may provide at least subjective improvements. Further studies examining ICD, including LCIG‐related mechanisms such as reductions in pulsatile dopaminergic stimulation, are also needed.

The effect of device‐aided therapies on caregiver burden has become a focus of research given its practical importance. Improvement in caregiver burden with LCIG might relate to improved mobility, which reduces the need for physical care,^50^ and improvement in nonmotor phenomenon (eg, sleep disturbances, ICD).^54^ Finally, research on device discontinuation is of significant practical value to both physicians and patients/caregivers. Complications prompting LCIG discontinuation are more common than those for DBS but generally less serious, and the ability to easily discontinue LCIG after repeated complications or lack of efficacy may be a feature of this device‐aided therapy.

Overall, treatment selection should consider individual patient circumstances with input from caregivers. Our review suggests LCIG appears favorable for addressing both motor and nonmotor symptoms of APD. Effects of LCIG on caregiver burden and nonmotor symptoms, including impulsivity, should also be taken into account. In addition, a shorter duration of disease may affect the response to LCIG treatment,^80^ but lifelong cost‐effectiveness should be considered at the same time. The main limitations of this review are related to the quality of the evidence of included studies, lack of consistency in reporting among similar studies, and the decision to limit our search to a single database. Limitations of the current evidence also include a lack of studies directly comparing different alternatives. Despite difficulties in conducting such studies, greater consistency in trial design and reporting may make indirect comparisons and pooling of results more reliable. Further, although the underlying mechanisms that explain the benefits of different devices are known to some extent, few studies have specifically examined this aspect of treatment. On a positive note, later studies have correlated benefits and outcomes with baseline patient characteristics, which should give practical assistance to clinicians in relation to treatment selection.

## Author Roles

T.T. and G.O. drafted and revised the manuscript. S.S., T.H., J.F., and Y.S. reviewed the manuscript and provided input. N.H. supervised this project. All authors approved the final version to be published.

## Financial Disclosures

Dr. Tsunemi was funded by grants from the Japan Society for the Promotion of Science, Grant‐in‐Aid for Scientific Research, and received speaker honoraria from Otsuka Pharmaceutical, Takeda Pharmaceutical Co. Ltd., and UCB Japan. Dr. Oyama was funded by Grant‐in‐Aid for Scientific Research and Novartis Pharma K.K., and received speaker honoraria from Medtronic, Boston Scientific, Otsuka Pharmaceutical Co. Ltd., Sumitomo Dainippon Pharma Co. Ltd., Eisai Co., Ltd., Takeda Pharmaceutical Company Ltd., Kyowa Hakko Kirin Co. Ltd., and AbbVie, Inc. Dr. Saiki was funded by grants from the Japan Society for the Promotion of Science, Grant‐in‐Aid for Scientific Research, and received speaker honoraria from Otsuka Pharmaceutical Co. Ltd., Sumitomo Dainippon Pharma Co. Ltd., Kyowa Hakko Kirin Co. Ltd., and Takeda Pharmaceutical Co. Ltd. Dr. Hatano was funded by grants from Sumitomo Dainippon Pharma Co. Ltd., Eisai Co. Ltd., the Setsuro Fujii Memorial, the Osaka Foundation for Promotion of Fundamental Medical Research, Japan Agency for Medical Research and Development (grant number 19 dm0107156), and received speaker's honoraria from Sumitomo Dainippon Pharma Co. Ltd., Takeda Pharmaceutical Co. Ltd., Kyowa Hakko Kirin Co. Ltd., Novartis Pharma K.K., Sanofi, Eisai Co. Ltd., and Otsuka, along with grant support. Dr. Fukae has nothing to declare. Dr. Shimo was funded by grants from the Japan Society for the Promotion of Science, Grant‐in‐Aid for Scientific Research, and received speaker honoraria from Medtronic, Boston Scientific, Otsuka Pharmaceutical, Takeda Pharmaceutical Co. Ltd., Sumitomo Dainippon Pharma Co. Ltd., Novartis Pharma K.K., MSD, FP Pharmaceutical Corporation, Kyowa Hakko Kirin Co. Ltd., and AbbVie, Inc. Dr. Hattori was funded by grants from the Japan Society for the Promotion of Science, the Ministry of Education Culture, Sports, Science and Technology, Japan, the Health Labour Sciences Research Grant, the Japan Agency for Medical Research and Development, AbbVie GK, FP Pharmaceutical Corporation Co., Sumitomo Dainippon Pharma Co. Ltd., and Eisai Co. Ltd.; and received speaker honoraria and payment for services on advisory boards from Kyowa Hakko Kirin Co. Ltd., Sumitomo Dainippon Pharma Co. Ltd., Takeda Pharmaceutical Co. Ltd., Otsuka Pharmaceutical Co. Ltd., AbbVie GK, Eisai Co. Ltd., FP Pharmaceutical Corporation Co., Mitsubishi Tanabe Pharma Co., Otsuka Pharmaceutical Co. Ltd., Novartis Pharma K.K., MSD, Nihon Medi‐Physics, Bristol‐Myers Squibb Company, Ono Pharmaceutical Co. Ltd., EA Pharma Co. Ltd., Asahi Kasei Medical Co. Ltd., and Chugai Pharma Manufacturing Co. Ltd.

## Supporting information


**Table S1** Summary of included articles
