# Supplementary material for: Intrajejunal Infusion of Levodopa/Carbidopa for Advanced Parkinson's Disease: A Systematic Review
Source: Mov Disord. 2021 Apr 25;36(8):1759–71. doi: 10.1002/mds.28595 (PMC9290931; doi:10.1002/mds.28595)
Supplement: Supplementary file 1 — Table S1 Summary of included articles [file MDS-36-1759-s001.docx]

# Supplementary Material

**Supplementary Table 1.** Summary of included articles

| **#** | **Reference** | **Title** | **Study type** | **N** | **Quality (1-5)** | **Limitations** | **Main relevance** |
| --- | --- | --- | --- | --- | --- | --- | --- |
| 1 | Antonini, 2016^22^ | Effect of levodopa-carbidopa intestinal gel on dyskinesia in advanced Parkinson's disease patients | Post hoc analyses of 12-week, randomized, double-blind study and 54-week open-label study | 169 | 2 | Exploratory, post hoc findings | Motor complications |
| 2 | Antonini, 2017^12^ | Levodopa-carbidopa intestinal gel in advanced Parkinson's: Final results of the GLORIA registry | Open-label, prospective/retrospective, observational study | 375 | 3 | Partially retrospective leading to missing data | Motor complications; non-motor symptoms |
| 3 | Artusi, 2020^69^ | Beyond 10 years of levodopa intestinal infusion experience: Analysis of mortality and its predictors. | Open-label, retrospective, longitudinal observational study | 98 | 3 | Open-label, lack of comparator, potential for missing data | Discontinuation |
| 4 | Bajenaru, 2016^60^ | The effect of levodopa-carbidopa intestinal gel infusion long-term therapy on motor complications in advanced Parkinson's disease: a multicenter Romanian experience | Open-label, retrospective observational study | 113 | 3 | Open-label, lack of comparator, potential for missing data, short duration | Motor complications; Optimal timing: initiation |
| 5 | Bohlega, 2015^13^ | Levodopa-carbidopa intestinal gel infusion therapy in advanced Parkinson’s disease: single middle eastern center experience | Open-label, prospective observational study | 20 | 3 | Open-label, lack of comparator, small size | Motor complications |
| 6 | Buongiorno, 2015^20^ | Long-term response to continuous duodenal infusion of levodopa/carbidopa gel in patients with advanced Parkinson disease: The Barcelona registry | Observational, prospective, multicenter registry study | 72 | 3 | Open-label, lack of comparator or control group | Motor complications |
| 7 | Busk, 2012^62^ | Long-term 24-h levodopa/carbidopa gel infusion in Parkinson's disease | Retrospective analysis of medical records (case series) | 21 | 4 | Open-label, lack of comparator, potential for missing data, small size | Optimal timing: administration duration |
| 8 | Caceres-Redondo, 2014^37^ | Long-term levodopa/carbidopa intestinal gel in advanced Parkinson’s disease | Long-term, retrospective, open-label observational study | 29 | 3 | Open-label, lack of comparator, potential for missing data, small size | Effect on QoL; complications |
| 9 | Catalan, 2018^47^ | Improvement of impulse control disorders associated with levodopa-carbidopa intestinal gel treatment in advanced Parkinson's disease. | Open-label, prospective observational study | 62 | 3 | Open-label design, lack of control group | Non-motor symptoms: impulse control |
| 10 | Chang, 2016^72^ | Intraduodenal levodopa-carbidopa intestinal gel infusion improves both motor performance and quality of life in advanced Parkinson's disease. | Open-label, prospective observational study | 15 | 3 | Open-label design, lack of control group, small size | Motor complications; Effect on QoL |
| 11 | Ciurleo, 2018^50^ | Assessment of Duodopa effects on quality of life of patients with advanced Parkinson's disease and their caregivers | Open-label, prospective observational study | 12 | 3 | Open-labe, lack of control group, small size | Effect on QoL; Effect on caregiver burden |
| 12 | Constantin, 2020^70^ | Levodopa-carbidopa intestinal gel infusion therapy discontinuation: a ten-year retrospective analysis of 204 treated patients | Retrospective, open-label, observational study | 204 | 3 | Open-label, lack of comparator, potential for missing data | Discontinuation |
| 13 | Cruse, 2018^23^ | 24-hour levodopa-carbidopa intestinal gel may reduce troublesome dyskinesia in advanced Parkinson's disease | Case series | 12 | 4 | Open-label, lack of comparator, potential for missing data, small size | Optimal timing: administration duration |
| 14 | De Fabregues, 2017^14^ | Long-term safety and effectiveness of levodopa-carbidopa intestinal gel infusion | Open-label, prospective, observational study | 37 | 3 | Open-label design, lack of control group, small size | Motor complications; Non-motor symptoms; Effect on ADL; Effect on caregiver burden |
| 15 | Ehlers, 2020^51^ | Levodopa infusion in Parkinson's disease: Individual quality of life | Prospective, open-label, observational study | 13 | 3 | Open-label design, lack of control group, small size, lack of validation of scales | Effect on QoL |
| 16 | Fabbri, 2019^75^ | Levodopa/carbidopa intestinal gel infusion and weight loss in Parkinson’s disease | Prospective, open-label, observational study | 44 | 3 | Open-label, lack of control group | Complications |
| 17 | Fabbri, 2019^32^ | Long-term effect of levodopa-carbidopa intestinal gel on axial signs in Parkinson's disease | Open-label, retrospective observational study | 49 | 3 | Open-label design, lack of control group, potential for missing data | Motor complications: freezing of gait |
| 18 | Fasano, 2012^43^ | Intrajejunal levodopa infusion in advanced Parkinson's disease: long-term effects on motor and non-motor symptoms and impact on patient's and caregiver's quality of life | Open-label, retrospective observational study | 14 | 3 | Open-label design, lack of control group, potential for missing data | Motor complications; Non-motor symptoms, including sleep; Effect on QoL |
| 19 | Fernandez, 2018^15^ | Long-term safety and efficacy of levodopa-carbidopa intestinal gel in advanced Parkinson's disease. | Open-label, prospective, observational study | 262 | 3 | Open-label, lack of control group | Motor complications; Non-motor symptoms; Effect on ADL; Discontinuation |
| 20 | Foltynie, 2013^52^ | Impact of Duodopa on quality of life in advanced Parkinson's disease: a UK case series. | Case series | 12 | 4 | Open-label, lack of control group, small size, potential for missing data | Effect on QoL |
| 21 | Jugel, 2013^76^ | Neuropathy in Parkinson's disease patients with intestinal levodopa infusion versus oral drugs. | Open-label, prospective, comparative observational study | 30 | 3 | Open-label, lack of baseline data, correlations only suggestive | Complications |
| 22 | Juhasz, 2017^16^ | Levodopa/carbidopa intestinal gel can improve both motor and non-motor experiences of daily living in Parkinson's disease: An open-label study | Open-label, prospective observational study | 34 | 3 | Open-label design, lack of control group | Non-motor symptoms, including sleep; Effect on QoL |
| 23 | Kruger, 2017^38^ | An observational study of the effect of levodopa–carbidopa intestinal gel on activities of daily living and quality of life in advanced Parkinson’s disease patients | Open-label, prospective, observational study | 64 | 3 | Open-label design, lack of control group, reliance on self-report | Non-motor symptoms; Effect on QoL; Effect on ADL |
| 24 | Lang, 2016^64^ | Integrated safety of levodopa-carbidopa intestinal gel from prospective clinical trials | Post hoc analysis of 4 prospective, multicenter phase 3 studies | 425 | 3 | Limitations of original open-label studies | Discontinuation |
| 25 | Lew, 2015^65^ | Initiation and dose optimization for levodopa-carbidopa intestinal gel: Insights from phase 3 clinical trials | Post hoc analysis of open-label study and double-blind trial | 354 | 3 | Inability of direct comparison, small size of double-blind trial | Optimal timing: treatment initiation; Discontinuation |
| 26 | Lopiano, 2019^17^ | Motor and non-motor outcomes in patients with advanced Parkinson's disease treated with levodopa/carbidopa intestinal gel: final results of the GREENFIELD observational study | Post-marketing observational study | 145 | 3 | Missing data among retrospectively assessed patients, no correction for dose, confounding from other treatment | Motor complications; Non-motor symptoms, including impulse control and sleep; Effect on QoL; Effect on caregiver burden |
| 27 | Marano, 2019^27^ | Complex dyskinesias in Parkinson patients on levodopa/carbidopa intestinal gel. | Retrospective, cross-sectional survey and retrospective, longitudinal case-control study | 208/49 | 3 | Open-label, lack of control group, lack of levodopa levels and motor diaries, potential for missing data | Motor complications |
| 28 | Meloni, 2017^26^ | Diphasic dyskinesias during levodopa-carbidopa intestinal gel (LCIG) infusion in Parkinson's disease | Case report | 33 | 4 | Open-label, small size | Motor complications |
| 29 | Merola, 2016^73^ | Advanced therapies in Parkinson's disease: Long-term retrospective study | Open-label, retrospective observational study | 60 | 3 | Open-label, lack of control group, potential for missing data, assessment method for dyskinesia | Motor complications; Effect on ADL |
| 30 | Merola, 2011^24^ | Comparison of subthalamic nucleus deep brain stimulation and Duodopa in the treatment of advanced Parkinson's disease | Cohort-control study | 40 | 3 | Open-label, confounding from heterogeneity among measures and patients | Motor complications |
| 31 | Merola, 2016^77^ | Peripheral neuropathy associated with levodopa-carbidopa intestinal infusion: a long-term prospective assessment. | Open-label, prospective, observational study | 33 | 3 | Open-label, lack of control group | Complications |
| 32 | Moes, 2020^71^ | Predictors of time to discontinuation of levodopa-carbidopa intestinal gel infusion: a retrospective cohort study | Open-label, retrospective cohort study | 98 | 3 | Open-label design, lack of control group, potential for missing data, lack of differentiation between permanent and temporary discontinuation | Discontinuation |
| 33 | Morales-Briceño, 2019^63^ | Long-term safety and efficacy of 24-hour levodopa-carbidopa intestinal gel in Parkinson's disease | Open-label, retrospective cohort study comparing 16-hour and 24-hour infusions | 99 | 3 | Lack of blinding and randomization between comparator groups, potential for missing data | Optimal timing: administration duration |
| 34 | Murata, 2018^18^ | Safety and efficacy of levodopa-carbidopa intestinal gel: results from an open-label extension study in Japanese, Korean and Taiwanese patients with advanced Parkinson's disease | Open-label, prospective, observational extension study | 27 | 3 | Open-label, lack of control group, small size, selection bias from entry criteria | Motor complications; effects on QoL |
| 35 | Olanow, 2014^10^ | Continuous intrajejunal infusion of levodopa-carbidopa intestinal gel for patients with advanced Parkinson's disease: a randomized, controlled, double-blind, double-dummy study | Double-blind, double-dummy, randomized study | 71 | 2 | No formal evaluation of blinding, short duration did not permit evaluation of long-term complications or efficacy | Motor complications |
| 36 | Poewe, 2019^66^ | Levodopa-carbidopa intestinal gel monotherapy: GLORIA registry demographics, efficacy, and safety | Post hoc analysis of a 24-month, multinational observational registry | 356 | 3 | Open-label, post hoc nature, retrospective design leads to missing data, real-world switching leads to selection bias | Motor complications; Non-motor symptoms; Discontinuation |
| 37 | Poewe, 2019^21^ | Levodopa-carbidopa intestinal gel in a subgroup of patients with dyskinesia at baseline from the GLORIA Registry | Post hoc analysis of a 24-month, multinational observational registry | 375 | 3 | Open-label, post hoc nature, retrospective design leads to missing data, real-world switching leads to selection bias | Motor complications; Non-motor symptoms; Effect on ADL; Effect on QoL |
| 38 | Ray Chaudhuri, 2019^39^ | Burden of non-motor symptoms in Parkinson's disease patients predicts improvement in quality of life during treatment with levodopa-carbidopa intestinal gel | Post hoc analysis of a 24-month, multinational observational registry | 233 | 3 | Open-label, post hoc nature, retrospective design leads to missing data, real-world switching leads to selection bias | Non-motor symptoms; Effect on QoL |
| 39 | Regidor, 2017^25^ | Duodenal levodopa infusion for long-term deep brain stimulation-refractory symptoms in advanced Parkinson disease | Open-label, prospective observational study | 40 | 3 | Open-label design, lack of control group, potential for missing data, small sample size in some groups | Motor complications; Non-motor symptoms: effect on sleep; Discontinuation |
| 40 | Regidor, 2019^61^ | Impact of disease duration in effectiveness of treatment with levodopa-carbidopa intestinal gel and factors leading to discontinuation | Open-label, retrospective observational study | 177 | 3 | Open-label, post hoc nature, retrospective design leads to missing data, possibility of confounding variables | Discontinuation |
| 41 | Rispoli, 2017^78^ | Peripheral neuropathy in 30 duodopa patients with vitamins B supplementation | Open-label, prospective observational study | 30 | 3 | Open-label, lack of control group, small size | Complications |
| 42 | Rispoli, 2018^30^ | Levodopa/carbidopa intestinal gel infusion therapy: focus on gait and balance | Open-label, prospective observational study | 15 | 3 | Open-label, lack of control group, small size | Motor complications: axial symptoms and gait |
| 43 | Sahlstrom, 2018^53^ | Workforce participation and activities in Parkinson's disease patients receiving device-aided therapy | Open-label, retrospective observational study | 67 | 3 | Open-label, potential for missing data, recall bias, selection bias may make results more favorable | Effect on ADL |
| 44 | Santos-Garcia, 2012^74^ | Long-term exposure to duodenal levodopa/carbidopa infusion therapy improves quality of life in relation especially to mobility, activities of daily living, and emotional well-being | Open-label, prospective observational study | 11 | 3 | Open-label, lack of control group, small size | Effect on QoL; Effect on ADL |
| 45 | Sensi, 2017^67^ | Which patients discontinue? Issues on levodopa/carbidopa intestinal gel treatment: Italian multicentre survey of 905 patients with long-term follow-up | Clinician survey | 905 | 5 | Recall bias of investigators, selection bias (only participating institutions), lack of uniform evaluation | Discontinuation |
| 46 | Sensi, 2014^29^ | Emerging issues on selection criteria of levodopa carbidopa infusion therapy: considerations on outcome of 28 consecutive patients | Open-label, prospective observational study | 28 | 3 | Open-label, lack of control group, small size | Motor complications, including axial symptoms and gait; Optimal timing: initiation |
| 47 | Slevin, 2015^11^ | Long-term safety and maintenance of efficacy of levodopa-carbidopa intestinal gel: an open-label extension of the double-blind pivotal study in advanced Parkinson's disease patients | Open-label extension of double-blind pivotal study | 62 | 3 | Open-label, lack of control group, small size | Motor complications |
| 48 | Standaert, 2018^41^ | Systematic evaluation of levodopa-carbidopa intestinal gel patient-responder characteristics | Open-label, prospective observational study | 39 | 3 | Open-label, lack of control group, small size | Non-motor symptoms; Effect on QoL; Optimal timing: initiation |
| 49 | Standaert, 2017^40^ | Effect of levodopa-carbidopa intestinal gel on non-motor symptoms in patients with advanced Parkinson's disease | Post hoc analysis of open-label, phase 3 study | 324 | 3 | Open-label design, lack of control group, post hoc nature | Motor complications |
| 50 | Tessitore, 2018^54^ | Caregiver burden and its related factors in advanced Parkinson's disease: data from the PREDICT study | Cross-sectional, epidemiologic study | 126 | 4 | Cross-sectional design excludes comparison with baseline, heterogeneous distribution of disease stage, exclusion criteria | Effect on caregiver burden |
| 51 | Todorova, 2017^46^ | Infusion therapies and development of impulse control disorders in advanced Parkinson disease: clinical experience after 3 years’ follow-up | Open-label, prospective observational study | 19 | 3 | Open-label, lack of comparator group, small size | Impulse control |
| 52 | Wang, 2018^19^ | Levodopa-carbidopa intestinal gel in parkinson's disease: a systematic review and meta-analysis | Systematic review and meta-analysis | 384 | 1 | Significant heterogeneity of included trials, small sample size, differences in control groups and lack of true placebo may underestimate effect of LCIG | Motor complications |
| 53 | Zibetti, 2018^31^ | Effects of intestinal levodopa infusion on freezing of gait in Parkinson disease | Open-label, retrospective observational study | 32 | 3 | Open-label design, lack of control group, potential for missing data, lack of specific scales for freezing of gait measurement | Motor complication: axial symptoms and gait |
| 54 | Zibetti, 2013^44^ | Sleep improvement with levodopa/carbidopa intestinal gel infusion in Parkinson disease | Open-label, prospective observational study | 12 | 3 | Open-label, lack of comparator group, short follow-up period, lack of objective measures of sleep | Sleep |
| 55 | Zibetti, 2013^81^ | Long-term duodenal levodopa infusion in Parkinson's disease: a 3-year motor and cognitive follow-up study | Open-label, prospective observational study | 12 | 3 | Open-label, lack of comparator group | Non-motor symptoms, including sleep |
| 56 | Zibetti, 2014^68^ | Levodopa/carbidopa intestinal gel infusion in advanced Parkinson's disease: a 7-year experience | Open-label, retrospective observational study | 59 | 3 | Open-label design, lack of control group, potential for missing data | Effect on QoL; Discontinuation |

Abbreviations: ADL, activities of daily living; QoL, quality of life
